# Prevalence, influencing factors, and prediction model construction of anemia in ankylosing spondylitis based on real-world data: An exploratory study

**DOI:** 10.1371/journal.pone.0318332

**Published:** 2025-02-05

**Authors:** Yifan Gong, Kun Yang, Zhaoyang Geng, Hongxiao Liu

**Affiliations:** 1 Guang’anmen Hospital, China Academy of Chinese Medical Sciences, Beijing, China; 2 Beijing University of Chinese Medicine, Beijing, China; Bursa Ali Osman Sonmez Oncology Hospital, TÜRKIYE

## Abstract

**Objective:**

This study aimed to explore the prevalence and influencing factors of anemia in patients with ankylosing spondylitis (AS) using real-world data and to construct a predictive model for anemia in AS.

**Methods:**

In November 2023, we accessed the database from China Rheumatoid Arthritis Registry of Patients with Chinese Medicine (CERTAIN). Clinical data of AS collected from the CERTAIN between March 2022 and September 2023 were analyzed. Demographic information, clinical assessment scales, and laboratory test results of the patients were collected. According to the anemia diagnostic criteria established by the World Health Organization (WHO) in 2018, patients were divided into anemia group and non-anemia group. Statistical analyses were performed using SPSS 25.0 software, including *χ*^*2*^ tests, independent sample t-tests to compare differences between the two groups, and multivariate stepwise logistic regression analysis to explore the influencing factors of anemia in AS. The predictive efficacy of the model was evaluated by plotting receiver operating characteristic (ROC) curves. Calibration was assessed through the Hosmer-Lemeshow goodness-of-fit test, and a calibration curve was plotted to comprehensively evaluate the predictive capability of the model.

**Results:**

A total of 251 patients were included in this study, among which 58 cases had anemia (23.1%). There were significant differences in gender, ossification, C-reactive protein (CRP), erythrocyte sedimentation rate (ESR) indicators, and clinical assessment scale results between the two groups *(P < 0.05).* The results of multivariate stepwise logistic regression analysis showed that female gender, underweight, ossification, abnormal CRP and ESR were independent risk factors for anemia in AS *(P < 0.05)*. Based on the results of multivariate stepwise logistic regression analysis, a predictive model for anemia in AS was established as Logit(P) = −5.02 + 2.041 × gender −1.11 × BMI(body mass index) category + 1.103 × ossification category + 0.942 × CRP category + 1.476 × ESR category. The ROC curve analysis showed that the area under the curve of the model for predicting anemia in AS was 0.857 (95% CI: 0.808 ~ 0.906). The Omnibus test of model coefficients yielded *χ*^*2*^ = 85.265, *P* < 0.001. The Hosmer-Lemeshow test showed *χ*^*2*^ = 7.005, *P* = 0.536 (*P* > 0.05).

**Conclusion:**

Analysis of real-world AS diagnosis and treatment data showed that the prevalence of anemia in Chinese AS was 23.1%. The occurrence of anemia was closely related to female gender, underweight, ossification, and abnormal CRP and ESR. The logistic model constructed based on these indicators for predicting the risk of anemia in AS demonstrated good efficacy.

## Introduction

Ankylosing spondylitis (AS) is a chronic autoimmune disease characterized primarily by chronic and progressive inflammation of the sacroiliac joints and spinal stiffness. The global prevalence of ankylosing spondylitis ranges from 0.1% to 1.4% [1], with an estimated prevalence in China of around 0.3%, with a male-to-female ratio of 2 to 4 to 1, and onset typically occurring between 15 and 40 years of age [2]. Despite its high prevalence and disability rates, the etiology and pathogenesis of AS remain unclear, and there is a lack of curative measures, severely impacting patients’ quality of life and prognosis.

Treatment of AS is a lengthy process, and throughout the patient’s entire lifespan, they may undergo treatment with non-steroidal antiinflammatory drugs (NSAIDs), corticosteroids, tumor necrosis factor-α(TNF-α) inhibitors, while also facing the inconvenience and psychological stress caused by the disease. Rheumatic immune system diseases are prone to accompanying hematologic abnormalities, with anemia being a common occurrence. Studies have reported that anemia is a common complication in rheumatic disease patients, which is related to the pathogenesis of autoimmune diseases [3]. Foreign studies have shown that anemia of varying degrees is common in patients with AS [4].

The reasons for decreased hemoglobin(Hb) levels in AS may be multifaceted, including gastrointestinal bleeding caused by long-term NSAID use, congenital Hb defects, concomitant ulcerative colitis or Crohn’s disease, radiation, or drug toxicity. There are differences in the incidence and prognosis of anemia among patients receiving different treatment regimens. For example, an Italian survey found anemia prevalence of 15% among AS using biologics [5], and after excluding anemia caused by other reasons, symptoms disappeared in 82% of patients after TNF-α treatment, a viewpoint corroborated by other studies [6,7]. In a cohort study in 2023, the proportion of patients receiving TNF-α treatment was 23% in the non-anemia group, while it was 16% in the anemia group [8].

China is a developing country, with anemia prevalence accounting for approximately 8.7% in the general population. Regrettably, there is limited research by Chinese clinicians and researchers on anemia in AS. We searched Chinese literature and did not find relevant reports on the prevalence and influencing factors of anemia in AS, and there is currently no definitive evidence regarding the incidence, influencing factors, and relationship with disease activity of anemia in Chinese AS. Clarifying the prevalence of anemia in Chinese AS will help in selecting optimal treatment strategies. This study aims to explore the prevalence and influencing factors of anemia in Chinese AS, analyze the impact of anemia on the disease condition of patients, investigate various factors influencing the prevalence of anemia, thus providing better guidance for clinical treatment and comprehensively improving patients’ quality of life.

## Objectives and methods

### Study subjects

This study is based on the China Rheumatoid Arthritis Registry of Patients with Chinese Medicine (CERTAIN). In November 2023, we accessed the database from CERTAIN. We collected data from the CERTAIN database from patients with AS treated in ten hospitals across China from March 2022 to September 2023. The participating hospitals were the Guang’anmen Hospital of China Academy of Chinese Medical Sciences (Beijing), Xiyuan Hospital of China Academy of Chinese Medical Sciences (Beijing), China-Japan Friendship Hospital (Beijing), Beijing Hospital of Traditional Chinese Medicine of Capital Medical University (Beijing), First Affiliated Hospital of Tianjin University of Traditional Chinese Medicine (Tianjin), People’s Liberation Army 81 Hospital (Hebei), First Affiliated Hospital of Anhui University of Chinese Medicine (Anhui), Affiliated Hospital of Liaoning University of Traditional Chinese Medicine (Liaoning), Affiliated Hospital of Shandong University of Traditional Chinese Medicine (Shandong), and Southwest Hospital of Army Military Medical University (Chongqing), including outpatient and inpatient AS departments of rheumatology and immunology. Written informed consent for study participation was obtained from all participants. This study was approved by the Ethics Committee of Guang’anmen Hospital, China Academy of Chinese Medical Sciences (2022-108-KY).

### Diagnostic and inclusion criteria

The diagnosis of AS was based on the revised New York criteria for AS established in 1984 [9] and the classification criteria for axial spondyloarthritis (SpA) recommended by the Assessment of SpondyloArthritis International Society (ASAS) in 2009 [10] were used as diagnostic criteria for AS. Inclusion criteria were as follows: (1) meeting the AS diagnostic criteria; (2) age 15 years or older; (3) voluntary participation in the clinical study and willingness to sign the informed consent form. Exclusion criteria were as follows: (1) patients with other rheumatic immune-related diseases; (2) pregnant or lactating women; (3) patients with severe infections within 1 month; (4) patients with incomplete or duplicate medical record information.

### Collection of clinical case information

A standardized information collection form was designed, and trained researchers collected patient information. Basic information collected for study subjects included gender, age, disease duration, height, weight, medical history, family history, etc. Laboratory test results included hemoglobin, erythrocyte sedimentation rate (ESR), C-reactive protein (CRP), and other inflammatory markers. Disease clinical assessment scales used included Ankylosing Spondylitis Disease Activity Score-CRP (ASDAS-CRP), ASDAS-ESR, Bath Ankylosing Spondylitis Function Index (BASFI), Bath Ankylosing Spondylitis Measurement Index (BASMI), Patient Global Assessment (PGA), nocturnal pain visual analogue scale (VAS), Assessment in SpondyloArthritis international Society Health Index (ASAS-HI), Functioning in the Treatment of Chronic Illnesses-Fatigue Inventory (FACIT-F), and Depression Anxiety Stress Scales (DASS-21).

### Definition of observational variables

The Anemia is diagnosed according to the World Health Organization (WHO) criteria established in 2018 [11], with hemoglobin (Hb) levels below 130 g/L for males aged 15 and above, and below 120 g/L for females aged 15 and above.

The Ectopic ossification refers to calcification changes occurring in non-calcified tissues, resulting in new bone formation in soft tissues. The Modified Stoke Ankylosing Spondylitis Spine Score (mSASSS) [12] was used to assess the progression of spinal ossification in AS.

The BMI less than 18.5 is classified as underweight, BMI between 18.5 and 24.9 is classified as normal, and BMI equal to or greater than 25 is classified as overweight or obese.

According to WHO classification standards, individuals aged 44 or younger are classified as young, while those aged 45 and older are classified as middle-aged or elderly.

The CRP levels were measured using immunoturbidimetric assays, with results greater than 10 mg/L considered abnormal.

The ESR was measured using the Westergren method, with results greater than 15 mm/h for males or greater than 20 mm/h for females considered abnormal.

The ASDAS [13,14] is an indicator used to assess disease activity in AS. It is categorized into four activity categories, including disease remission (ASDAS < 1.3), low disease activity (1.3 ≤ ASDAS < 2.1), high disease activity (2.1 ≤ ASDAS ≤ 3.5), and very high disease activity (ASDAS > 3.5). The formulas for calculating ASDAS-CRP and ASDAS-ESR scores are provided.

The BASFI [15] evaluates the patient’s functional ability in anatomical activities (bending, stretching, rotation, standing, turning, climbing stairs) and coping ability in daily life. It consists of 10 items scored using a 10 cm visual analog scale, with higher scores indicating worse joint function.

The BASMI [16] is used to assess the spinal mobility of patients. It evaluates joint function by measuring ear-wall distance, cervical spine rotation degrees, lumbar spine lateral bending, lumbar spine mobility, and ankle distance. Higher scores indicate worse joint function.

The FACIT-F [17] includes 13 questions, with responses scored on a Likert scale from 0 to 4. The sum of scores is the fatigue index, ranging from 0 to 52, with lower scores indicating higher levels of fatigue.

The ASAS-HI [18] is a questionnaire based on the ASAS for assessing the health status of patients with SpA. It measures the functional status of 17 items in SpA patients, with higher scores indicating a greater impact of the disease on the overall health status of patients.

The DASS-21 [19] is a self-reported questionnaire designed to assess depression, anxiety, and stress. Each aspect contains 7 items, with responses scored on a Likert scale from 0 to 3. Scores for depression, anxiety, and stress are calculated separately, with higher scores indicating worse emotional states.

The nocturnal pain score [20] is used to assess nighttime pain using a 10 cm visual analog scale, with scores ranging from 0 to 10, where higher scores indicate greater pain severity.

The PGA [21] is an overall assessment of disease activity by patients using a VAS over the past week, with scores ranging from 0 to 10, where higher scores indicate worse overall condition.

### Statistical methods

Statistical analysis was conducted using SPSS 25.0 software. Count data were represented by frequency and proportion (%). Group comparisons were made using the *χ*^*2*^ test. The Shapiro-Wilk (S-W) test was used to assess the normality of continuous variables. Variables following a normal distribution were described as mean ± standard deviation($\bar{x}\pm s$), and differences between groups were analyzed using an independent sample t-test. Variables not following a normal distribution were described as median (interquartile range)*[M(P25,P75)]*, and differences were analyzed using the Kruskal-Wallis test or the Mann-Whitney U test. Anemia status was set as the dependent variable, while other factors were treated as covariates. Stepwise regression analysis was first conducted to screen independent variables. Variables with a univariate analysis *P* < 0.2 were included in a multivariate logistic regression analysis to establish the model. The predictive performance of the model was evaluated using the area under the receiver operating characteristic (ROC) curve (AUC). Calibration was assessed through the Hosmer-Lemeshow goodness-of-fit test, and a calibration curve was plotted to comprehensively evaluate the predictive capability of the model. Differences were considered statistically significant at *P < 0.05*.

## Results

### Comparison of characteristics between anemic and non-anemic groups

A total of 251 patients were included in this study, with 58 cases of anemia, yielding an incidence rate of 23.1% (58/251). There was a statistically significant difference in gender between the anemic and non-anemic groups *(P < 0.05).* There were statistically significant differences in ossification, CRP, ESR, ASDAS-CRP, fatigue, and anxiety between the anemic and non-anemic groups *(P < 0.05)* (Table 1).

**Table 1:** Comparison of characteristics between anemic and non-anemic groups.

Variable	Total (n = 251)	non-anemic group (n = 193)	anemic group (n = 58)	*χ* ^*2*^	*P-*value
**Sex**				1.496	0.001
** Male**	205 (81.4)	166 (86.0)	39 (67.2)		
** Female**	46 (18.3)	27 (14.0)	19 (32.8)		
**Age, year**				3.459	0.063
** ≤ 44**	180 (71.7)	144 (74.6)	36 (62.1)		
** ≥ 45**	71 (28.3)	49 (25.4)	22 (37.9)		
**BMI,kg/m** ^**2**^				3.241	0.198
** < 18.5**	13 (5.2)	10 (5.2)	3 (5.2)		
** 8.5-24.9**	144 (57.4)	105 (54.4)	39 (67.2)		
** ≥ 25.0**	94 (37.4)	78 (40.4)	16 (27.6)		
**HLA-B27,positive**	152 (60.6)	117 (60.6)	35 (60.3)	0.001	0.970
**Family History,yes**	56 (22.3)	41 (21.2)	15 (25.9)	0.549	0.459
**Extra articular** **manifestation,yes**	68 (27.1)	52 (26.9)	16 (27.6)	0.009	0.923
**Ossification, yes**	74 (29.5)	42 (21.8)	32 (55.2)	23.946	0.000
**CRP (mg/L)**				28.291	0.000
** ≤10**	178 (70.9)	153 (79.3)	25 (43.1)		
** >10**	73 (29.1)	40 (20.7)	33 (56.9)		
**ESR (mm/h)**				37.688	0.000
** normal**	144 (57.4)	131 (67.9)	13 (22.4)		
** abnormal**	107 (42.6)	62 (32.1)	45 (77.6)		
**ASDAS-CRP**				26.239	0.000
** < 1.3**	49 (19.5)	46 (23.8)	3 (5.2)		
** 1.3 ≤ ASDAS -CRP < 2.1**	78 (31.1)	64 (33.2)	14 (24.1)		
** 2.1 ≤ ASDAS-CRP ≤ 3.5**	102 (40.6)	74 (38.3)	28 (48.3)		
** >3.5**	22 (8.8)	9 (4.7)	13 (22.4)		
**FACIT-F**				5.096	0.024
** little or no fatigue**	153 (61.0)	125 (64.8)	28 (48.3)		
** significant fatigue**	98 (39.0)	68 (35.2)	30 (51.7)		
**Depression**				1.272	0.259
** normal**	211 (84.1)	165 (85.5)	46 (79.3)		
** abnormal**	40 (15.9)	28 (14.5)	12 (20.7)		
**Anxiety**				4.049	0.044
** normal**	197 (78.5)	157 (81.3)	40 (69.0)		
** abnormal**	54 (21.5)	36 (18.7)	18 (31.0)		
**Stress**				0.235	0.628
** normal**	229 (91.2)	177 (91.7)	52 (89.7)		
** abnormal**	22 (8.8)	16 (8.3)	6 (10.3)		

n: number of case; BMI:Body Mass Index; Human Leukocyte Antigen B27(HLA-B27); CRP:C-reactive protein; ESR:erythrocyte sedimentation rate; ASDAS-CRP: Ankylosing Spondylitis Disease Activity Score-C-reactive protein; FACIT-F: Functional Assessment of Chronic Illness Therapy-Fatigue.

### Comparison of multidimensional clinical assessment of patients

The mean values of ASDAS-ESR, ASDAS-CRP, BASFI, BASMI, ASAS-HI, depression score, anxiety score, stress score, nighttime pain VAS, and PGA were higher in the anemic group compared to the non-anemic group. The median FACIT-F score was lower in the anemic group than in the non-anemic group. Statistically significant differences were observed in ASDAS-ESR, ASDAS-CRP, BASFI, BASMI, ASAS-HI, depression score, anxiety score, stress, nighttime pain VAS, and PGA between the anemic and non-anemic groups *(P < 0.05)* (Table 2).

**Table 2:** Comparison of multidimensional clinical assessment of patients.

Variable	non-anemic group (n = 193)	anemic group (n = 58)	*Z*	*P-*value	95%CI
**ASDAS-ESR**	1.916 (1.244,2.511)	2.730 (2.354,3.539)	−6.549	0.000	−1.295 ~ −0.771
**ASDAS-CRP**	1.974 (1.259,2.649)	2.579 (1.921,3.498)	−4.450	0.000	−1.083 ~ −0.500
**BASFI**	0.40 (0.00,2.25)	2.00 (0.50,5.55)	−3.901	0.000	−2.041 ~ −0.451
**BASMI**	2.00 (0.00,5.00)	6.00 (2.00,8.00)	−5.083	0.000	−3.511−1.693
**FACIT-F**	42.00 (36.00,44.00)	38.50 (34.00,44.00)	−2.848	0.004	0.920 ~ 5.167
**ASAS-HI**	0.00 (0.00,1.00)	3.00 (0.00,9.25)	−4.639	0.000	−4.763 ~ −1.673
**Depression**	0.00 (0.00,6.00)	4.00 (0.00,8.00)	−3.654	0.000	−4.209 ~ −0.772
**Anxiety**	2.00 (0.00,6.00)	4.00 (2.00,8.00)	−2.623	0.000	−3.186 ~ −0.477
**Stress**	2.00 (0.00,8.00)	6.00 (1.50,12.00)	−2.134	0.033	−3.477 ~ 0.254
**Nocturnal pain VAS**	1.00 (0.00,3.00)	3.00 (1.00,5.00)	−3.488	0.001	−1.861 ~ −0.453
**PGA**	4.00 (1.50,6.00)	6.00 (4.00,7.00)	−4.543	0.000	−2.508 ~ −1.017

n: number of case; CI, confidence interval; ASDAS-ESR: Ankylosing Spondylitis Disease Activity Score-Hematological Sedimentation; ASDAS-CRP: Ankylosing Spondylitis Disease Activity Score-C-reactive protein; BASFI: Bath Ankylosing Spondylitis Functionality Index; BASMI: Bath Ankylosing Spondylitis Measurement Index; FACIT-F: Functioning in Chronic Illness Treatment-Fatigue Scale; ASAS-HI: Ankylosing Spondylitis Health Index; VAS: visual analogue scale; PGA: Patient Global Assessment.

### Logistic regression analysis of factors influencing anemia in AS

Using anemia as the dependent variable (1 = yes, 0 = no), variables with *P* < 0.2 in the univariate analysis were selected as covariates for multiple logistic regression analysis. The results showed that female gender (OR = 7.695, 95% CI: 3.033 ~ 19.518), being underweight (OR = 0.33, 95% CI: 0.164 ~ 0.662), ossification (OR = 3.013, 95% CI: 1.307 ~ 6.945), abnormal CRP (OR = 2.565, 95% CI: 1.086 ~ 6.061), and abnormal ESR (OR = 4.376, 95% CI: 1.854 ~ 10.332) were independent risk factors for anemia in ankylosing spondylitis patients, with statistically significant differences *(P <  0.05)* (Table 3).

**Table 3:** Multivariable stepwise logistic regression analysis of anemia in AS.

Variable	β	SE	Wald *χ*^*2*^	*P-*value	OR(95%CI)
**Sex**	2.041	0.475	18.46	0.000	7.695 (3.033 ~ 19.518)
**BMI**	−1.110	0.356	9.733	0.002	0.33 (0.164 ~ 0.662)
**Ossification**	1.103	0.426	6.704	0.010	3.013 (1.307 ~ 6.945)
**CRP**	0.942	0.439	4.610	0.032	2.565 (1.086 ~ 6.061)
**ESR**	1.476	0.438	11.345	0.001	4.376 (1.854 ~ 10.332)
**Constant**	−5.020	0.926	29.409	0.000	0.007

SE, standard error; OR, odds ratio; CI, confidence interval; BMI, body mass index; CRP, C-reactive protein; ESR, erythrocyte sedimentation rate.

Assignment: Sex: 1 =  Male, 2 =  Female; Age: 0 = (≤44); 1 = (≥45); BMI: 0 = (<18.5 kg/m²), 1 = (18.5–24.9 kg/m²), 2 = (≥25.0 kg/m²); Ossification: 0 = No, 1 = Yes; CRP: 0 = Normal, 1 = Abnormal; ESR: 0 = Normal, 1 = Abnormal.

### Prediction model and efficacy analysis of anemia in AS

**Fig 1:**
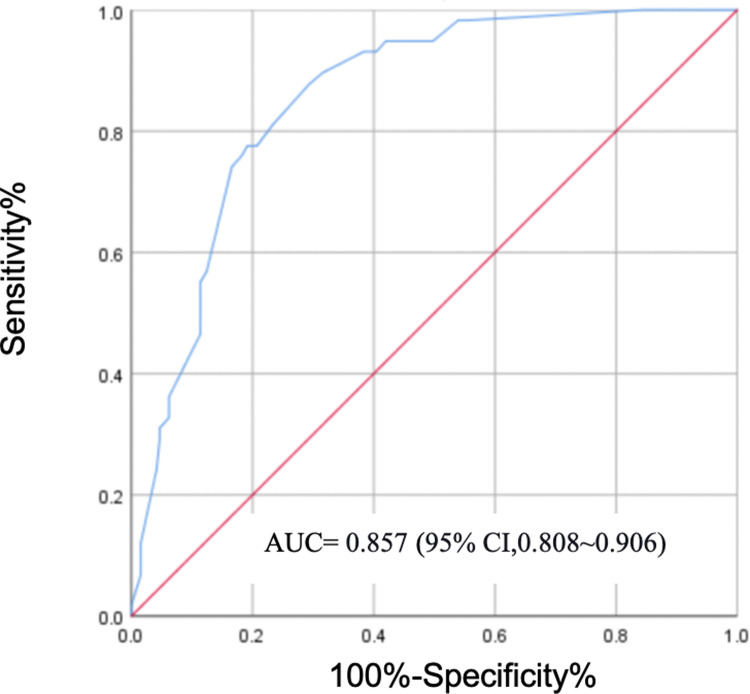
Receiver Operating Characteristic (ROC) curve of the predictive model. ROC: Receiver Operating Characteristic; AUC: area under the receiver operating characteristic (ROC) curve.

Based on the results of multiple logistic regression analysis, the five aforementioned risk factors were included in the prediction model for anemia in AS. The equation for the prediction model was Logit(P) =  −5.02 + 2.041 × gender −1.11 × BMI category + 1.103 × ossification category + 0.942 × CRP category +  1.476 × ESR category. Using the model value as the predictive indicator, ROC analysis was performed. The prediction model constructed with these five factors demonstrated high predictive efficacy for anemia in AS: ROC-AUC (95% CI) was 0.857 (0.808 ~ 0.906); the optimal cutoff value was 0.190, with a sensitivity of 0.879 and specificity of 0.805, resulting in an overall prediction accuracy of 0.857 (Fig 1).

**Fig 2:**
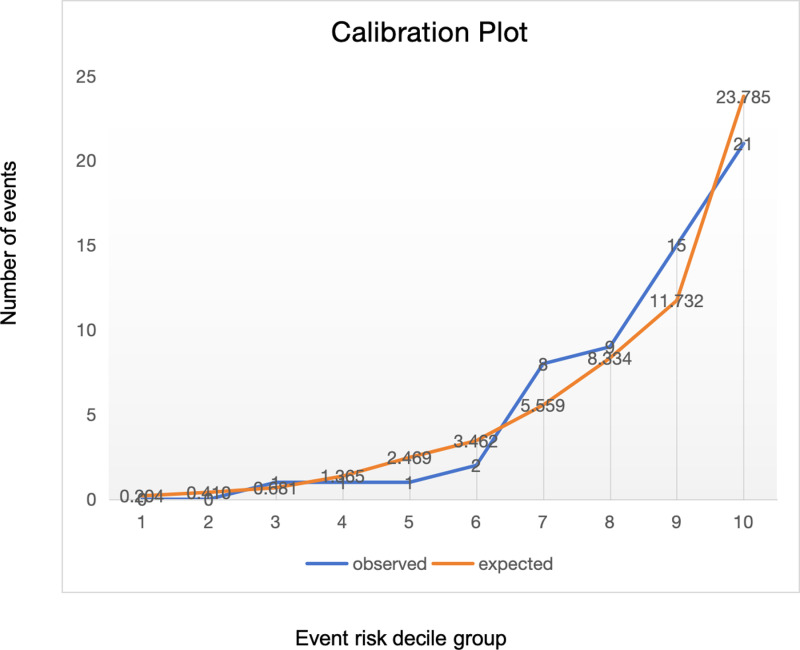
Calibration plot.

The Omnibus test of model coefficients yielded *χ*^*2*^ = 85.265, *P* < 0.001, indicating that the model is statistically significant. The Hosmer-Lemeshow test showed *χ*^*2*^ = 7.005, *P* = 0.536 (*P* > 0.05), suggesting that there is no statistically significant difference between the predicted and observed values, indicating a good model fit. We plotted a calibration curve to visualize the calibration results of the predictive model (Fig 2).

## Discussion

AS, as a complex autoimmune disease, is characterized by inflammation, ossification, and bone destruction, with its incurable and protracted course severely affecting patients’ quality of life. Studies from different countries have reported the occurrence of anemia in AS, but the reported prevalence of anemia in AS varies among studies. A clinical series study of AS in Mexico found that 78% of subjects under 16 years old and 47.5% of adults had hemoglobin levels below the normal range [22]. In 2020, a study in Ukraine involving 118 AS showed that 28.8% of patients developed anemia [23]. Research on the prevalence of AS-associated anemia in China has not been reported to date. In this study, the prevalence of anemia in AS was 23.1%, higher than the 8.7% anemia rate in adult residents of China (with a male anemia rate of 4.2% and a female anemia rate of 13.2%) [24], but slightly lower than reported in other countries.

Anemia is a serious global public health issue, and reducing anemia has been included as one of the six global nutrition goals by the World Health Assembly [25]. The harm caused by anemia is multifaceted. Anemia can lead to compromised immune function, impaired growth and development, and diminished cognitive abilities, making patients prone to fatigue, reducing productivity, and lowering overall quality of life, thereby increasing the risk of mortality. Anemia also imposes a significant economic burden on families and society [26].

Our study found that AS with anemia had significantly higher BASDAI, ASDAS indices, CRP, and ESR compared to non-anemic patients, indicating a close correlation between disease activity, inflammation, and anemia. This suggests that the occurrence of anemia in AS is closely related to the high activity of the inflammatory process and is also a marker of disease activity. A study team from the German Cancer Research Center observed that anemia is associated with inflammation, which can cause damage to hematopoietic function and have irreversible effects on functional hematopoietic stem cells [27]. As a chronic inflammatory disease, controlling inflammation in AS should be emphasized throughout the course of the disease. In addition, compared to non-anemic patients, anemic patients had poorer physical function, patient-reported outcomes, and psychological status, indicating that the overall health status of anemic patients with AS is lower. Therefore, clinical practitioners should pay more attention to AS with concurrent anemia.

Anemia prevalence among AS in China was significantly higher than that in the general population, exerting a notable impact on patients’ health and quality of life. The multifactorial analysis conducted in this study revealed that gender, BMI, ectopic ossification, CRP, and ESR are independent risk factors influencing anemia in AS. Our findings suggest that female gender, lower BMI, ectopic ossification, and higher levels of inflammatory markers are associated with an increased susceptibility to anemia. Utilizing these factors, a predictive model was constructed for assessing the risk of anemia in AS, demonstrating favorable predictive performance. Consistent with our findings, previous investigations by international scholars have reported a prevalence of anemia among female AS as high as 32%, compared to 8% in males, which aligns with our study results [28]. Furthermore, existing research indicates significantly elevated levels of inflammatory markers (CRP, ESR) in anemic AS compared to non-anemic individuals, corroborating the findings of our study [23]. For female AS with ectopic ossification, lower BMI, and heightened inflammatory activity, it is recommended to screen for concomitant anemia and analyze the variations in anemia pathogenesis to facilitate tailored therapeutic interventions.

Diverse treatment regimens also influence the occurrence of anemia. Commonly used medications for treating ankylosing spondylitis, such as non-steroidal anti-inflammatory drugs or immunosuppressants, may contribute to anemia. Studies have shown that the likelihood of anemia occurrence is lower in patients receiving TNF-α treatment, possibly due to TNF-α’s role in alleviating inflammation [8]. Therefore, TNF-α treatment may be considered as a priority for AS with concurrent anemia to control inflammation, reduce disease activity, and subsequently lower the incidence of anemia.

This study is the first to report the prevalence of anemia in Chinese AS and has yielded some important findings. However, there are two main limitations to note. Firstly, the inclusion of patients from selected hospitals resulted in a small sample size, with significant differences in sample sizes among different hospitals, leading to limited geographical representativeness and potential bias in the results. Secondly, clinical diagnostic and treatment data were not included as possible observed variables affecting the etiology of anemia, limiting further analysis of the causes of anemia prevalence.

In conclusion, the prevalence of anemia in Chinese AS is significantly higher than that in the general population, substantially impacting patients’ disease progression and quality of life. We recommend conducting further related studies to clarify the factors and mechanisms influencing the occurrence of anemia in AS, providing a basis for developing diagnostic and therapeutic strategies for AS-associated anemia.

## Conclusion

In this study, the prevalence of anemia in AS was 23.1%. Female gender, low BMI, ossification, elevated ESR, and abnormal CRP levels were identified as independent risk factors for anemia in AS. Moreover, the predictive model constructed based on these indicators exhibited good efficacy for predicting the risk of anemia in AS.
